# Who Wants to Be an Intrapreneur? Relations between Employees’ Entrepreneurial, Professional, and Leadership Career Motivations and Intrapreneurial Motivation in Organizations

**DOI:** 10.3389/fpsyg.2017.02041

**Published:** 2017-11-22

**Authors:** Kim-Yin Chan, Moon-Ho R. Ho, Jeffrey C. Kennedy, Marilyn A. Uy, Bianca N. Y. Kang, Olexander S. Chernyshenko, Kang Yang T. Yu

**Affiliations:** ^1^Nanyang Business School, Nanyang Technological University, Singapore, Singapore; ^2^School of Social Sciences, Nanyang Technological University, Singapore, Singapore; ^3^Massey Business School, Massey University, Auckland, New Zealand

**Keywords:** entrepreneurship, professionalism, leadership, innovation, intrapreneurship, motivation, human capital, scale development

## Abstract

This paper reports an empirical study conducted to examine the relationship between employees’ Entrepreneurial, Professional, and Leadership (EPL) career motivations and their intrapreneurial motivation. Using data collected from 425 working adults in the research/innovation and healthcare settings, we develop a self-report measure of employee intrapreneurial motivation. We also adapt an existing self-report measure of E, P, and L career motivations (previously developed and used with university students) for use with working adult organizational employees. Confirmatory factor analysis indicate that E, P, and L motivations and intrapreneurial motivation can be measured independently and reliably, while regression analyses show that the employees’ E, P, and L motivations all contribute to explaining variance in their intrapreneurial motivation. Individuals with high E, P, and L motivational profiles are also found to have the highest intrapreneurial motivation scores, while those low on E, P, and L motivations have the least intrapreneurial motivation. Our findings suggest that the potential for intrapreneurship is not unique to only entrepreneurial employees. Instead, one can find intrapreneurs among employees with strong leadership and professional motivations as well. We discuss the findings in the context of generating more research to address the challenges of talent management in the 21st century knowledge economies where there is greater career mobility and boundarylessness in the workforce.

## Introduction

In a knowledge-based, innovation-driven economy, human capital plays a major role in an organization’s success (Takeuchi et al., 2007; Crook et al., 2011). This is because people form the basis for the intellectual capital of any organization and its ability to innovate (Teece, 2000; Subramaniam and Youndt, 2005). Organizational interest has therefore grown in relation to the concept of “intrapreneurs” – akin to entrepreneurs working in organizations (see Antoncic and Hisrich, 2001) – and, to the concept of Innovative Work Behavior (Farr and Ford, 1990; de Jong and Den Hartog, 2010).

Given the importance of human capital for organizational innovation, it is useful to consider how one can conceptualize and measure the motivational potential of an organization’s human capital to innovate, or the motivational aspect of an organization’s intrapreneurial capacity. To that end, a recently proposed Entrepreneurship, Professionalism, and Leadership (EPL) framework (Chan et al., 2012) seems particularly relevant as it has been shown to be related to boundaryless career attitudes – that various writers have argued are particularly relevant to knowledge workers operating in the innovation sector (e.g., Saxenian, 1996; DeFillippi et al., 2006; Inkson, 2008). The EPL framework also facilitates examination of whether the motivation to innovate within one’s current organization is primarily found among those who possess high entrepreneurial motivation, or if one can also expect to find intrapreneurial motivation among those motivated for leadership or professional work/roles, independent of their entrepreneurial aspirations. Addressing this question will benefit discussions on the differences between entrepreneurs and intrapreneurs. More importantly, it will also provide a new approach for thinking about an organization’s innovative capacity based on the microfoundations (cf. Felin and Foss, 2005) of the organization’s human capital, thereby complementing macro-level theories of organizational innovation (e.g., absorptive capacity by Cohen and Levinthal, 1990). Such an approach would also respond to Ployhart et al.’s (2014) call for better understanding of multilevel relationships between human capital resources (or capacities) at the individual- and unit-levels and unit- or organizational-level outcomes.

Our empirical study has the following goals: (1) to develop a measure of employee intrapreneurial motivation; (2) to adapt and provide initial validation of Chan et al.’s (2012) self-report measures of career motivations (previously developed and used with university students) for use with working adult organizational employees; and (3) to explore the question “who wants to be an intrapreneur?” by examining the relationship between employees’ EPL career motivations and their intrapreneurial motivation. Specifically, we examine if intrapreneurial motivation is primarily correlated with entrepreneurial motivation (if one were to treat intrapreneurs essentially as employees with entrepreneurial qualities), or, if E, P, and L motivations are all correlated and add incremental validity to the prediction of intrapreneurial motivation. The latter finding would indicate that intrapreneurs can be found not only among entrepreneurial employees but also among those with deep technical/professional interests and those who are motivated for leadership roles in the organization.

### Intrapreneurial Motivation: Unique to the Entrepreneurial or Also Found among Leader-Managers and/or Deep, Technical Professionals?

Two separate literatures have attempted to address the question: Who is most motivated to pursue innovation in an organization? These are: the study of “Intrapreneurship” in the field of entrepreneurship, and, the study of Innovative Work Behavior in the field of organizational behavior.

Schumpeter (1934) first focused early attention of entrepreneurship researchers on the important role of entrepreneurs – whom he saw as highly individualistic and self-directed individuals who were motivated to challenge convention or tradition and were thus key to the “creative destruction” needed for innovation in the economy. Half a decade later, Pinchot (1985) popularized the concept of “intrapreneurship” by describing intrapreneurs as “dreamers who do. Those who take hands-on responsibility for creating innovation of any kind, within a business” (p. ix). Today, the trait/person-based view of entrepreneurship is thus most typically associated with individuals who strive to innovate for themselves while owning much of the business they start-up; while intrapreneurship or “corporate entrepreneurship” refers to organizational employees who focus on innovation and creativity and seek to transform “a dream or an idea into a profitable venture by operating within the organizational environment” (p. 149; Pinchot, 1985).

Hisrich (1990) considered intrapreneurship a “hybrid form of entrepreneurship.” Motivationally and behaviorally, he emphasized the similarities between entrepreneurs and intrapreneurs: “Intrapreneurs, like entrepreneurs, take new ideas and develop solid, functioning, and, it is hoped, profitable businesses. Intrapreneurs possess the same entrepreneurial spirit as entrepreneurs” (p. 209). Others have emphasized the contextual differences between entrepreneurship and intrapreneurship. For example, Nicolaidis and Kosta (2011) observed: “The main difference between entrepreneur and intrapreneur is that entrepreneurs innovate for themselves, as they mostly own much of the business they start-up, while intrapreneurs innovate on behalf of an established organization in which they may have no equity within the company or only a small percentage” (p. 1469). The common theme underlying intrapreneurship and entrepreneurship is clear: Innovation.

Writing in the field of entrepreneurship, Antoncic and Hisrich (2001) attempted to establish the core dimensions of intrapreneurship as follows: (1) a new-business-venturing dimension (referring to the pursuit and entry into new businesses related to the firm’s current products or markets); (2) an innovativeness dimension (referring to the creation of new products, services, and technologies); (3) a self-renewal dimension (referring to strategy reformulation, reorganization, and organizational change), and (4) a proactiveness dimension (referring to top management initiative and risk-taking, and competitive aggressiveness, and boldness). They later defined intrapreneurship as “a process that goes on inside an existing firm, regardless of its size, and leads not only to new business ventures but also to other innovative activities and orientations such as development of new products, services, technologies, administrative techniques, strategies, and competitive postures” (p. 498; Antoncic and Hisrich, 2001).

Separately, from an organizational behavior perspective, de Jong and Den Hartog (2010) attempted to conceptualize and measure employee Innovative Work Behavior without specific reference to the concepts of entrepreneurship or intrapreneurship. Recognizing Farr and Ford’s (1990) definition of Innovative Work Behavior as an individual’s behavior that aims to achieve the initiation and intentional introduction (within a work role, group, or organization) of new and useful ideas, processes, products, or procedures, they proposed and developed measures to operationalize employee Innovative Work Behavior in terms of four dimensions: (1) idea exploration, (2) idea generation, (3) idea championing, and (4) idea implementation.

While some have written on the “motivation to innovate” (e.g., Perry et al., 1992; Fernandez and Pitts, 2011), there is no existing direct measure of intrapreneurial motivation in the social scientific literature. For the purpose of this study, we decided to develop a self-report measure of employee intrapreneurial motivation with reference to both Antoncic and Hisrich (2001) and de Jong and Den Hartog’s (2010) dimensions. Given the centrality of innovation to both intrapreneurship and entrepreneurship, we gave particular emphasis to Antoncic and Hisrich’s innovation-oriented dimensions. This measure would then serve as a “criterion” construct for addressing the question: “how are E, P, and L motivations related to intrapreneurial motivation?,” which would also help us determine if E, P, and L could collectively represent the dimensions of an organization’s human capital capacity to innovate.

Having a measure of intrapreneurial motivation would also allow us to examine the distinctiveness of employee entrepreneurial motivation in relation to intrapreneurial motivation. Specifically, we sought to establish if intrapreneurial motivation only correlated significantly with entrepreneurial motivation, or, if E, P, and L motivations were all correlated and added to predicting intrapreneurial motivation among employees.

### Entrepreneurship, Professionalism, and Leadership As Dimensions of Motivation at Work

In a chapter entitled “Careers and the wealth of nations,” Kanter (1989) proposed that entrepreneurship, professionalism, and bureaucracy (or managerialism) represented three different logics of work that could help one understand the relationship between individual careers and macro-level economic outcomes. Put simply, entrepreneurs were motivated to work because of a market-driven logic or desire to create value from opportunities, professionals were driven by a logic of expertise and reputation, while the bureaucrats or managers were motivated by a desire to organize and control resources. Separately, Schein (1971, 1978) proposed a conceptual scheme for linking individuals and their careers to their organizations. He argued that careers could be conceptualized as moving in three dimensions: (1) vertically or up a hierarchy, (2) functionally within or across specializations, and (3) centrality or the extent to which one was close to the core or distant (to the extent of being outside) the organization. In his conceptual scheme, Schein (1971) made it clear that people – the organization’s human capital – were the basis innovation and change in organizations; that it is through “individual influence” that change or innovation occurs.

Building on these multi-level theories and recognizing the increasingly boundaryless (Arthur, 1994) and protean (Hall, 1996) nature of careers in the 21st century, Chan et al. (2012) suggested that E, P, and L could be a way for individuals to think about the dimensions of boundaryless career space as a framework for their own career development. They showed that E, P, and L career aspirations (operationalized as E, P, and L motivations, efficacies, and intentions) could be measured independently and reliably in a large sample of over 10,000 university students. They also found that individuals who were high in EPL motivations were most boundaryless and self-directed (or protean) in their career attitudes. They therefore proposed that EPL could be a multidimensional framework for individuals to visualize their careers as vectors moving in a boundaryless, three-dimensional career space.

To the extent that employees in an organization vary in their motivation for entrepreneurship, professionalism, and leadership, it is possible that EPL could also serve as a multidimensional framework for conceptualizing the motivational potential of an organization’s human capital. Individual decisions (such as selection, project assignments, or performance management) could be tailored to support or develop individual motivations. Similarly, the composition of teams could include consideration of the relative importance of EPL motivations in light of team tasks. At the organizational level, processes such as workforce planning and talent management could incorporate information on the firm’s EPL portfolio alongside traditional information on skills and experience. Such an approach may be particularly relevant in knowledge-based, innovation-driven economies where organizations need a mix of employees with deep technical/professional expertise and motivations, and also leadership skills and drive, and entrepreneurial instincts. We suggest that such a mix of qualities contributes to the organization’s potential to innovate, that employees’ E, P, and L motivations independently yet additively contribute to predicting intrapreneurial motivation.

### Research Aim

To reiterate, our overarching aim is to address the question “who wants to be an intrapreneur?” by examining the relationship between employees’ EPL career motivations and their intrapreneurial motivation. To do so, the first goal is to adapt and develop Chan et al.’s (2012) self-report measures of EPL motivation (previously developed and used only with university students) for use with working adults or employees in organizations. The purpose of this effort is to determine if E, P, and L motivations could be measured independently and reliably in a working adult context, and verify whether Chan et al.’s (2012) finding that individuals with high-EPL motivational profile were also highest in boundaryless mindset and protean (self-directed) career attitudes can be replicated using the working adult context. The second goal is to develop a measure of intrapreneurial motivation and to establish if this was a distinct construct – independent from entrepreneurial motivation. With initial evidence for construct validity for the intrapreneurial motivation scale, we then address the question “how are E, P, and L motivations related to employee intrapreneurial motivation?”: If we could show that employees’ E, P, and L motivations independently yet additively contributed to their intrapreneurial motivation, then this would support our idea of adopting EPL as a framework for assessing the capacity of an organization’s human capital for innovation or its “intrapreneurial potential.”

## Materials and Methods

### Sample

To achieve the goals of scale development and to examine the relationships between EPL and intrapreneurial motivation among organizational employees or working adults at the individual level of analysis, specific effort was made to recruit participants from different organizations and job-types (e.g., research versus administrative staff) to avoid range restriction in our overall sample. A total of 425 working adult employees volunteered to participate in our research survey; they were recruited from two different organizational/work settings where employee innovation was considered relevant: (1) 360 research scientists, technical and administrative staff (47% male, 53% female; 62.3% were aged between 20 and 35 years old, 27.1% were aged between 36 and 45 years old, 10.6% were aged above 45 years old; and mean working of 9.1 years, *SD* = 8.5 years) from a large public sector research organization were individually approached through recruitment posters and word-of-mouth; (2) 65 nurses from a public sector hospital in Singapore (28% male, 72% female; 23.1% were aged between 20 and 35 years old, 38.5% were aged between 36 and 45 years old, 38.5% were aged above 45 years old; mean working experience = 19.6 years, *SD* = 12.7 years) voluntarily responded to an invitation email from the researchers that was forwarded by their personnel department. All participants completed the survey online and acknowledged the informed consent form before beginning the survey in accordance to procedures approved by the Institutional Review Board. Respondents answered the questionnaire anonymously; most took 15–20 min to complete it online. Participants from the public sector research organization were offered and accepted monetary compensation for their participation. However, the nurses from the public sector hospital volunteered to participate without taking any compensation.

### Measures

#### EPL Motivation Scales for Working Adults

The EPL motivation measure used in the present study comprised 27 items that were further developed following a pilot study that was an initial attempt to adapt Chan et al.’s (2012) EPL scales (previously developed and used with university students) for use with working adult organizational employees. In that pilot study, 18 items were only slightly modified from the EPL measure developed by Chan et al. (2012; developed for use with university students) and administered to a total of 214 working adults (41% male; 59% female; 80% aged 21–40 years old with the remaining 20% above 40 years; average working experience of 10.4 years with a *SD* of 8 years) from healthcare, research, innovation and enterprise sectors. Confirmatory factor analyses showed that a three-factor model, corresponding to E motivation, P motivation, and L motivation, fit the data reasonably well (χ^2^ = 511.09, *df* = 132, χ^2^/*df* = 3.87, CFI = 0.87, RMSEA = 0.08, GFI^∗^ = 0.98), compared with the one factor model (χ^2^ = 1704.7, *df* = 135, χ^2^/*df* = 12.63, CFI = 0.45, RMSEA = 0.22, GFI^∗^ = 0.92) [^∗^ the specific GFI computed and reported in this paper is Gamma-hat (γ) by Maiti and Mukherjee (1990); see also Steiger (1989), which has been shown to be resistant to sample size, model complexity, and model misspecification (Fan and Sivo, 2007)].

Feedback from pilot study participants and organizations prompted us to revise further and add more items for our working adult EPL measure and to consider the research question in this study “how are E, P, and L motivations related to employee intrapreneurial motivation?” and to develop the intrapreneurial motivation scale for the present research. Hence, in the present study, most of the items from Chan et al.’s (2012) original scales (developed and used with university students) were further re-worded and some new items constructed to ensure greater relevance for use with working adults. Each subscale (Entrepreneurial, Professional, and Leadership motivation) now had nine items, resulting in a total of 27 items in the EPL measure used in the present study. Items were rated on a 5-point Likert scale, from 1 (strongly disagree) to 5 (strongly agree). Confirmatory factor analysis of data from the 425 working adults showed that the three 9-item subscales were independent from each other and from the intrapreneurial motivation scale. **Table 1** lists the EPL motivation items and their factor loadings based on a confirmatory factory analysis alongside the intrapreneurial motivation scale. All factor loadings reported in **Table 1** are statistically significant and with the exception of one noncalculative leadership motivation item with a loading of 0.36, all others meet Hair et al.’s (2010) recommended a rule of thumb that standardized factor loadings should be 0.5 or higher. Cronbach’s alpha item analysis showed that three subscales were reliable: 9-item Entrepreneurial motivation scale α = 0.87, 9-item Professional motivation scale α = 0.80, and 9-item Leadership motivation scale α = 0.84. Composite reliability [specifically, McDonald’s (1999) Omega] computed for the three scales was 0.91, 0.90, and 0.85, respectively.

**Table 1:** Items and standardized factor loadings for EPL motivation and intrapreneurial motivation scales.

	Factor loading
**Intrapreneurial motivation scale**	
I like convincing coworkers and management to support an innovative idea.	0.69
I would take a responsibility for development of new products and services.	0.81
I would be among the first to implement new ideas or processes in my work unit, because this is one of the best ways to make a real difference.	0.71
If asked by my organization to move to another district or abroad and set up new operations there, I would gladly accept this opportunity.	0.64
I enjoy coming up with new and practical ideas to expand the range of products and services that my organization can offer.	0.75
Making senior management enthusiastic about innovative ideas proposed in my work unit is one strategy I would use to make an impact and stay visible.	0.71
I feel a need to actively contribute to the development of new products and services.	0.82
I am interested in pursuing opportunities that create value for my organization.	0.74
I am the kind of person who always wonders how things can be improved in my organization.	0.76
**Entrepreneurial motivation scale**	
If my family or friends asked me to go into business, I would consider it favorably.	0.74
The rewards and satisfaction of starting and running a business far outweigh the risks and sacrifices needed.	0.61
I see working for myself as the best way to escape the rigidity and routines of organizations/companies.	0.55
Since young, I aspire to own a business.	0.65
I am the type of person that is best suited to be an entrepreneur.	0.88
I am the kind of person who constantly has ideas about new businesses.	0.78
I have always been taught in the value of starting a business (e.g., it provides jobs and helps the economy).	0.58
Starting and running my own company will allow me to derive the full reward of my own efforts and ideas.	0.68
This country needs more entrepreneurs and I feel obliged to “give it a go.”	0.78
**Professional motivation scale**	
I enjoy reading articles and attending courses that deepen or update my professional expertise.	0.63
If asked to teach in an advanced course or program in my specialty area, I would be honored to do it.	0.70
Being a respected professional will assure me of a steady income, prestige, and status in society.	0.57
I am best suited for professional jobs where I can make use of the knowledge I have gained in the past.	0.67
I feel that I have a responsibility to stay and excel in my current profession.	0.58
I care deeply about advancing and creating knowledge in my area of expertise (specialization).	0.74
The best way to increase my country’s competitiveness is for people like me to become highly skilled professionals in my industry.	0.61
My chosen profession will give me a comfortable life with acceptable prestige or status in society.	0.58
If I stick to being a profession in my industry, I am guaranteed to make a good living.	0.54
**Leadership motivation scale**	
When I agree to lead a group, I don’t seek advantages or special benefits.	0.50
I would agree to be a project leader whenever asked by my coworkers.	0.69
I don’t expect to get any privileges if I agree to lead or be responsible for a project.	0.36
I have always enjoyed leading others and would assume leadership roles whenever I could.	0.85
I am the kind of person who likes influencing and managing people more than doing anything else.	0.67
I would accept a managerial position if nominated by peers or senior management.	0.71
I am interested in leading groups even if there are no clear advantages for me.	0.68
If I am nominated to be in charge of a project or a group, I feel it is an honor and privilege to accept such a role.	0.79
I am definitely more of a leader by nature, so I am happy to assume leadership responsibilities whenever I can.	0.81

*N = 425. Confirmatory factor analysis with MLR estimation (maximum-likelihood estimation with parameter estimates robust to non-normality) in MPlus version 8.*

#### Intrapreneurial Motivation Scale

Taking reference from Antoncic and Hisrich’s (2001) four dimensions of intrapreneurship (i.e., new-business venturing, innovativeness, self-renewal, and proactiveness) and also de Jong and Den Hartog (2010) four dimensions of Innovative Work Behavior (i.e., idea exploration, generation, championing, implementation), we constructed nine statements that reflected individual interest and motivation to engage in intrapreneurial and innovative actions in their organization. Respondents were asked to indicate the extent they disagreed or agreed with each statement on a 5-point scale, from 1 (strongly disagree) to 5 (strongly agree). Confirmatory factor analysis of data from the 425 working adults showed that the 9-items were unidimensional (χ^2^ = 67.31, *df* = 27, χ^2^/*df* = 2.49, CFI = 0.96, RMSEA = 0.06, GFI^∗^ = 0.98) and distinct from the EPL motivation factors. **Table 1** lists the items and their factor loadings based on a confirmatory factory analysis alongside the EPL motivation items. All items were loaded 0.5 and above on the appropriate factor. Cronbach’s alpha internal consistency reliability and composite reliability for the 9-item scale were both good at α = 0.88 and McDonald’s Omega = 0.91.

#### Boundaryless Mindset and Protean Career Attitude Scales

To replicate Chan et al.’s (2012) finding that individuals with high-EPL motivational profile were also highest in boundaryless mindset and protean (self-directed) career attitudes, we included 5- and 6-items, respectively, from these two scales in Briscoe et al.’s (2006) measure. These were selected from those with the highest factor loadings in our pilot study of the EPL working adult scale in an attempt to reduce the overall length of the questionnaire. Respondents indicated on a 5-point scale the extent to which they felt the statements were true about them (1 = little or no extent, 5 = to a great extent). Examples of boundaryless mindset items were: “If development opportunities are not offered by my organization/company, I am the kind who would seek these out on my own,” and “Overall, I want a very independent, self-directed career.” Sample items for protean career attitude were: “I would enjoy working on projects with people across many organizations/companies” and “I would like tasks at work that require me to work beyond my own department.” A higher score on each of the subscales indicated a greater degree of each attitude. Confirmatory factor analyses showed that a measurement model with two factors provided very good fit to the data (χ^2^ = 124.59, *df* = 43, χ^2^/*df* = 2.90, CFI = 0.95, RMSEA = 0.07, GFI^∗^ = 0.97) compared to a single-factor fitted to all 11 items (χ^2^ = 351.77, *df* = 44, χ^2^/*df* = 8.00, CFI = 0.82, RMSEA = 0.13, GFI^∗^ = 0.88). Means, standard deviations, and Cronbach’s alpha statistics for the two sub-scales are presented in **Table 2**. Both scales had good Cronbach’s alpha reliabilities of 0.88 and 0.85, and McDonald’s Omega of 0.91 and 0.88 for the boundaryless mindset and protean scales, respectively.

**Table 2:** Scale means, standard deviations, reliabilities, and inter-scale correlations.

Variables	Number of items	*M*	*SD*	1	2	3	4	5	6
(1) Entrepreneurial motivation	9	3.11	0.69	**(0.87/0.91)**					
(2) Professional motivation	9	3.87	0.54	0.30	**(0.80/0.90)**				
(3) Leadership motivation	9	3.55	0.61	0.51	0.59	**(0.84/0.85)**			
(4) Intrapreneurial motivation	9	3.68	0.61	0.56	0.51	0.55	**(0.88/0.91)**		
(5) Boundaryless mindset	5	3.49	0.82	0.30	0.38	0.51	0.45	**(0.88/0.91)**	
(6) Protean career attitude	6	3.75	0.75	0.18	0.32	0.37	0.32	0.64	**(0.85/0.88)**

*N = 425. Diagonals in brackets indicate Cronbach’s alpha reliability/McDonalds Omega composite reliability for each scale. All correlations are significant at *p* < 0.001.*

## Findings

### Confirmatory Factor Analyses of EPL and Intrapreneurial Motivation Scales

As our survey response scales were ordinal in nature and because we observed minor skewness in the item-level distributions, we used MLR estimation (maximum-likelihood estimation with parameter estimates robust to non-normality) in MPlus version 8 (Muthén and Muthén, 1998–2017) for our confirmatory factor analyses to avoid any issues related to the violation of multivariate normality in maximum-likelihood estimation (Flora and Curran, 2004; Rhemtulla et al., 2012).

As our data were based on cross-sectional, self-report surveys, we checked and attempted to control for the threat of common method bias, first by fitting a single common-factor to all 36 EPL and intrapreneurial motivation items. We compared the fit of this model against that of the proposed 4-factor model (i.e., Harman’s test). Furthermore, we also examined a measurement model containing an unmeasured latent method factor in addition to the four-factor model (**Table 3**). Multigroup confirmatory factor analysis showed that the single-factor model generated poor fit with relative to the measurement model with four first-order factors, whereas the additional latent methods factor only improved model fit slightly. Configural, metric, and scalar invariance was also established across both organizational samples. The fit indices were comparable with the measurement model fit reported by Chan et al. (2012) for their EPL scales with university student data. Based on these findings, we can therefore conclude that common method bias was not a major threat to our findings (Podsakoff et al., 2003), and, that E, P, and L motivations could be measured independently of each other and with intrapreneurial motivation. This analysis provides initial evidence for construct validity of the focal scales in this study.^1^

**Table 3:** Combined and multigroup confirmatory factor analysis of EPL and intrapreneurial motivation scale items.

Model	χ^2^	*df*	*p*	χ^2^*/df*	RMSEA	CFI	GFI^∗^
**Single-factor model**							
Factor loadings freely estimated across samples	3754.85	1188	0.001	3.16	0.10	0.57	0.75
Invariant factor loadings across samples	3796.16	1223	0.001	3.10	0.10	0.57	0.75
Invariant factor loadings and error variances across samples	3945.06	1258	0.001	3.14	0.10	0.55	0.74
**Four-factor model**							
Factor loadings freely estimated across samples	2022.31	1176	0.001	1.72	0.04	0.89	0.90
Invariant factor loadings across samples	2076.96	1208	0.001	1.72	0.04	0.89	0.90
Invariant factor loadings and error variances across samples	2249.81	1240	0.001	1.81	0.04	0.87	0.88
**Four-factor model controlling for unmeasured latent methods factor**							
Factor loadings freely estimated across samples	1767.85	1104	0.001	1.60	0.04	0.92	0.92
Invariant factor loadings across samples	1945.81	1206	0.001	1.61	0.04	0.91	0.91
Invariant factor loadings and error variances across samples	2052.26	1237	0.001	1.66	0.04	0.90	0.90

*N = 425 comprising *n*_*1*_ = 360 research scientists, technical, and administrative staff from a large public sector research organization and *n*_*2*_ = 65 nurses from a public sector hospital. RMSEA, root mean square error of approximation; CFI, comparative fit index; GFI, gamma-hat (γ) goodness-of-fit index by Maiti and Mukherjee (1990).*

### Correlations and Regression of Intrapreneurial Motivation on EPL Motivations

**Table 2** summarizes the correlations among the various scales included in this study. We observed that the three EPL motivations were all moderately correlated with intrapreneurial motivation at between *r* = 0.51 and *r* = 0.56.

**Table 4** summarizes the findings of a stepwise regression where gender, age, and a dummy-coded variable indicating the two sub-samples were included as independent variables together with E, P, and L motivations to predict the intrapreneurial motivation. Gender, E, P, and L motivations all predicted the intrapreneurial motivation significantly and were retained in the final model. Together, they accounted for about 47% of the variance in intrapreneurial motivation. The variance inflation factor (VIF) statistic is close to 1, which indicates that multi-collinearity is not a concern in this regression analysis. This indicates that intrapreneurs can be found not only among entrepreneurially motivated employees but also among those with deep technical/professional interests and those who are motivated toward leadership roles in the organization.

**Table 4:** Results of stepwise regression analysis.

Predictors	DV = intrapreneurial motivation		
	β	*t*	VIF
Entrepreneurial motivation	0.36	8.47^∗∗∗^	1.37
Professional motivation	0.26	5.86^∗∗∗^	1.53
Leadership motivation	0.19	3.94^∗∗∗^	1.85
Gender	–0.12	–3.12^∗∗^	1.08

*R^*2*^ = 0.47, *F*(4,415) = 90.95, *p* < 0.001. ^∗∗^*p* < 0.01, ^∗∗∗^*p* < 0.001, *n* = 425, standardized regression coefficients are reported. Gender is dummy-coded, with 1 = female group and 0 = male group.*

### How Boundaryless/Protean Career Attitudes and Intrapreneurial Motivation Relate to EPL Profiles

To show the relationship between the EPL motivations and the 21st century career attitudes like boundaryless mindset and protean career attitudes, Chan et al. (2012) categorized their research participants into eight “profile” groups on the basis of whether their entrepreneurial, professional, and leadership motivation scores were above or below the mean obtained for 10,326 university participants. They then showed that individuals concurrently high in E, P, and L career motivations were also highest in boundaryless mindset and protean career attitudes, followed by those high in two out of the three EPL motivations. In contrast, individuals who were low on all three EPL motivations were lowest on these career attitudes.

**FIGURE 1:**
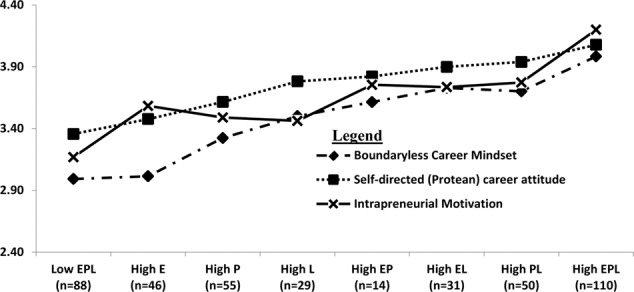
Mean values of boundaryless, protean career attitudes, and intrapreneurial motivation scale across eight EPL motivation groups.

Using the same approach, we created the eight EPL profile groups for our sample of 425 working adults and computed the mean scores on boundaryless mindset and protean career attitude for these eight profile groups. These means are presented in **Table 5** and plotted in **Figure 1**. We observe that the groups with the highest and lowest mean values on boundaryless mindset and protean career attitude were the high EPL and low EPL groups, respectively. This therefore replicates Chan et al.’s (2012) finding with our working-adult version of the EPL motivation scales, providing support for the validity of these measures adapted for use with working adults.

**Table 5:** Mean scores for boundaryless, protean career attitudes, and intrapreneurial motivation scales across eight EPL profiles.

Variable	Low EPL (*n* = 88)	High E (*n* = 46)	High P (*n* = 55)	High L (*n* = 29)	High EP (*n* = 14)	High EL (*n* = 31)	High PL (*n* = 50)	High EPL (*n* = 110)	One-way ANOVA		
									*F*-test	Tukey’s HSD	η^2^
Boundaryless career mindset	2.99	3.01	3.32	3.50	3.61	3.73	3.70	3.98	17.7^∗∗∗^	1 < 4,6,7,8; 2 < 6,7,8; 3,4 < 8	0.23
Protean (self-directed) career attitude	3.36	3.48	3.62	3.78	3.82	3.90	3.94	4.08	9.5^∗∗∗^	1 < 6,7,8; 2 < 7,8; 3 < 8	0.14
Intrapreneurial motivation	3.17	3.58	3.49	3.46	3.75	3.73	3.77	4.20	35.6^∗∗∗^	1 < 2,3,5,6,7,8; 2,3,4,5,6,7 < 8	0.38

*N = 425. Homogeneity of variance holds for all tests. η^*2*^ = effect size measure. All possible Tukey’s HSD tests were performed to test all possible pairwise comparisons among the eight groups for the three scales.*

In addition, we found that individuals with the highest and lowest mean values on intrapreneurial motivation were the high EPL and low EPL groups, respectively (see **Table 5** and **Figure 1**). One-way analysis of variance (homogeneity of variance holds across all comparisons) showed significant differences among the mean scores of the eight groups on all three measures. This suggests that EPL motivations additively relate to intrapreneurial motivation and reinforces our earlier conclusion that intrapreneurs can be found not only among entrepreneurially motivated employees.

## Discussion

This paper adapts Chan et al.’s (2012) EPL measures (previously developed for use with for university students) for use with working adults, and also introduces a new self-report measure for intrapreneurial motivation that may be used with organizational employees. More importantly, we show that E, P, and L motivations all correlate-with and significantly predict the motivation to innovate or intrapreneurial motivation, which suggests that intrapreneurship is not only unique to entrepreneurial employees, but that one can find intrapreneurs among employees with strong leadership and professional motivations as well.

### Theoretical Contributions

Given the importance of innovation as a dynamic capability for firms (Teece, 2016), this study makes an important contribution to the understanding of microfoundations of organizational competitive advantage. If we are to understand how organizational routines supporting intrapreneurship are developed and embedded in firms, we need to start with valid individual measures of relevant constructs. While there is further work to do in clarifying the functional relations among measures of intrapreneurial motivation at individual, unit, and organizational levels (Chan, 1998), our study has provided a basis from which to move forward. While the focus of this study has been on individual motivations, a valid individual-level measure opens up the possibility of creating profiles of group membership, and investigating how variations in such profiles are associated with group performance. What mix of leadership, professional, and entrepreneurial motivations seem most effective in different types of teams (e.g., teams responsible for new product development, change implementation, cross-functional problem-solving, departmental management, and the like)?

One of the core challenges facing innovative organizations is how to take knowledge stored at the individual level and make it available (and useable) at the organizational level (Schneckenberg et al., 2015). The dynamic capability framework (Teece, 2007) identifies microfoundations of innovative and adaptive capabilities as being located in managerial and organizational structures, systems, processes, and procedures. However, focusing at the level of structures and processes diverts attention away from the role of individuals in innovation. As Schneckenberg et al. (2015) note: “While learning and knowledge sharing take place in community conversations, the quality of conversations depends on cognitive and attitudinal prerequisites of community members” (p. 364). Our study highlights the importance of one attitudinal component (i.e., EPL motivation) in the context of firm innovation.

That E, P, and L motivations additively predicted intrapreneurial motivation corresponds to the multi-disciplinary notion of innovation. Drawing across domains (in our case, the broad domains of E, P, and L) is a defining element of innovation which is often described alongside processes of boundary crossing and integration of different disciplines, knowledge, and expertise (Tushman, 1977; Garud et al., 2013).

This study also helps to clarify discussions on the differences between intrapreneurs and entrepreneurs. First, we show that one can measure both entrepreneurial and intrapreneurial motivations independently among organizational employees. Second, the finding that E, P, and L motivations all correlate-with and significantly predict intrapreneurial motivation suggests that organizations should look for intrapreneurs not only among those with an entrepreneurial streak, but also among those with leadership and professional motivations. Moreover, our findings imply that intrapreneurial motivation applies to employees who are not only high in E-motivation but also in P- and L-motivations.

Our findings also help to further differentiate intrapreneurship from entrepreneurship. Studies of the personality of entrepreneurs have highlighted a positive relationship between risk-taking propensity and entrepreneurial intention (e.g., Zhao et al., 2010). Similarly, the cultural value of uncertainty avoidance has been shown to be relevant to engagement in entrepreneurial activities with Hofstede (2001, p. 164) noting that low uncertainty avoidance “implies a greater willingness to enter into unknown ventures.” Entrepreneurs carry a higher financial risk, given their higher levels of ownership in start-up businesses (Nicolaidis and Kosta, 2011), suggesting the need for a greater risk tolerance. In contrast, intrapreneurs have a greater degree of security, working for a salary within a supportive corporate structure. Our study suggests that high levels of P and L motivation are (within this supportive environment) associated with higher levels of intrapreneurial motivation. People with highly developed technical expertise whose tolerance for risk may lead them to avoid entrepreneurial pursuits are nevertheless motivated to innovate in intrapreneurial roles. This suggestion that innovative people may self-select into intrapreneurial or entrepreneurial roles is consistent with the arguments put forward by Felin et al. (2009) regarding self-selection of expert talent within and across organizations in the knowledge economy.

Finally, this study also extends Chan et al.’s (2012) person-centered application of EPL (for thinking about and developing their careers in boundaryless space) toward more organizational applications, e.g., the possibility of assessing unit-level human capital capacity for innovation from individual-level E, P, and L motivations and efficacies, and of team composition on the basis of E, P, and L motivations and efficacies/skills, etc. It suggests that the EPL framework may be relevant for conceptualizing organizational human capital capacity to innovate.

### Limitations and Future Research

Our study is not without limitations. First, because all our data were self-reported, common method bias could be a threat (Podsakoff et al., 2003). Thus, we used both Harman’s single-factor method and the unmeasured latent factor technique as statistical methods to examine this issue. Whereas these analyses show that common method bias did not seriously jeopardize our results, we also note that concerns have been raised about such statistical techniques. These include concerns that the unmeasured variable could reflect other types of variance due to unanticipated relationships between our constructs. Hence, we encourage future research to employ study design procedures such as temporal or psychological (e.g., using a cover story to reduce salience of linkage between measures) separation that would further alleviate such concerns (Podsakoff et al., 2012).

Second, we view our research as an initial step toward a more concerted effort to investigate the relatively unexplored phenomenon of intrapreneurial motivation within the context of work and careers. Consequently, further research is needed to continue to build on our initial validation efforts of the construct and its measure. Establishing construct validity is a continuous process (Hinkin, 1998; Shaffer et al., 2016), and we recommend that future research extend current efforts to establish discriminant validity between intrapreneurial and other work-related motivations [e.g., motivation to develop (Maurer et al., 2003) and prosocial behavior (Grant, 2008)]. More evidence is also needed to establish predictive validity with relevant workplace outcomes such as employee work engagement and performance (Christian et al., 2011).

Lastly, our context (i.e., employees from the public-sector) limits our study’s generalizability. Future research should consider other contexts including the private sector to extend the generalizability of our findings to other contexts.

### Applications

Schneckenberg et al. (2015) make the point that “sources for corporate innovation have become more dependent on high-level scientific and technological knowledge” (p. 356). Bendig et al. (in press) recognize this, and recommend that companies seeking to build dynamic capabilities in areas such as R&D need to invest training and information sharing around current technologies. This emphasis on deep technical knowledge is consistent with high levels of P motivation in the EPL framework. However, our study suggests that firms need to also consider complementary motivations, as both L and E make additional contributions to intrapreneurial motivation.

Scientific and technological expertise is central to innovation in knowledge firms (Schneckenberg et al., 2015), so enhancing the E and L motivation of high P technical experts will have a payoff in terms of overall intrapreneurial motivation. P motivation (the desire to continue learning and extending specialist knowledge) is important, because knowledge of firm-relevant technology is a vital precursor to understanding how new knowledge might be assimilated or exploited. Zucker et al. (1998) demonstrated the critical role of “scientist-entrepreneurs” (p. 291) in the nascent US biotechnology industry; enhancing the E motivation of technical experts to develop “scientist-intrapreneurs” could have similar impact within knowledge firms.

Leadership is also an important adjunct to P motivation. Motivation to lead is associated with a desire to bring about change, to challenge the status quo, and a willingness to influence others. Importantly, L motivation encompasses the willingness to accept the responsibilities and possible costs associated with leading (Chan and Drasgow, 2001). Antoncic and Hisrich (2001) identify different aspects of the intrapreneurship construct, but they all share the requirement that existing patterns, processes, products, or technologies are being challenged and renewed. Leadership is the process which moves good ideas out of the lab and into core business.

Given the newness of the EPL framework, there is no empirical research into the ability of training interventions to increase specific E, P, or L motivations. However, there is a relevant precedent in learned needs theory (McClelland, 1961). McClelland (1961) reported how high achievement motivation was associated with some aspects of entrepreneurial venture performance, and training programs have been shown to enhance achievement motivation (Miron and McClelland, 1979). This suggests that training interventions could benefit firms seeking to create higher and more pervasive levels of intrapreneurial motivation.

Our study informs organizations considering to launch intrapreneurship programs and workshops in their respective companies that intrapreneurship is not only for the select few who are non-conformists (i.e., those who exhibit entrepreneur-like qualities). As noted by Govindarajan and Desai (2013), “You already have natural intrapreneurs in your company. Some you know about, but most are hiding. These individuals are not always your top talent or the obvious rebels or mavericks.” Hence, intrapreneurship training and development should not be limited to those who are entrepreneurially inclined because there is no singular form of intrapreneurship, and equating intrapreneurs with entrepreneurs is a naïve supposition (Antoncic and Hisrich, 2001).

### Conclusion: Leveraging Human Capital for Innovation in an Era of Career Mobility and Boundarylessness

Felin et al. (2009) highlight a number of trends affecting the ability of knowledge firms to control the process of generating and sharing new knowledge. Several of these trends reflect shifts away from traditional organizational careers toward less structured employment relationships, yet firms are still reliant on people as “the source of knowledge, discretion, expertise and activity…accessing talent is the critical capability for generating competitive advantage in the knowledge economy” (p. 566). Taken together, these claims argue for the importance of understanding motivation at the individual level. Firms are increasingly reliant not only on the capacity and motivation of people to develop deep technical knowledge continually, but also to apply this in innovative ways and influence others to adopt new ideas. High levels of EPL motivation are associated with greater levels of intrapreneurial motivation, and provide an improved career motivation framework for assessing and developing the talent on which innovative firms rely.

## Ethics Statement

This study was carried out in accordance with the recommendations of the Institutional Research Board (IRB), Nanyang Technological University with informed consent from all subjects. The protocol was approved by the IRB, Nanyang Technological University, Singapore.

## Author Contributions

K-YC and M-HH were involved in the design of the study, data analysis, and manuscript writing. MU and OC were involved in the design of study and manuscript writing. BNYK assisted data collection and data analysis. JK and KYTY were involved in manuscript writing and revisions.

## Conflict of Interest Statement

The authors declare that the research was conducted in the absence of any commercial or financial relationships that could be construed as a potential conflict of interest. The reviewer AL and handling Editor declared their shared affiliation.

## Footnotes

1 We have also explored other variations of the measurement model to represent E, P, L, and intrapreneurial motivation including merging the intrapreneurial motivation factor with one of the E/P/L motivation factors, which lead to a three-factors representation. However, the four factors model remains as the best-fit model in terms of Chi-square statistic and other goodness of fit indices considered in this paper.
